# Use of standardised outcome measures among physiotherapists in French-speaking sub-Saharan Africa

**DOI:** 10.4102/sajp.v80i1.1981

**Published:** 2024-01-26

**Authors:** Abdoulaye Sawadogo, Emmanuel Segnon Sogbossi, Gauthier J. Everard, Toussaint Kpadonou, Charles Sèbiyo Batcho

**Affiliations:** 1 School of Physiotherapy, Faculty of Health Sciences, University of Abomey-Calavi, Cotonou, Benin; 2 University Clinic of Physical Medicine and Rehabilitation, Centre National Hospitalier Universitaire Hubert Koutoukou MAGA, Cotonou, Benin; 3 School of Rehabilitation Sciences, Faculty of Medicine, Université Laval, Quebec, Canada; 4 Centre Interdisciplinaire de Recherche en Réadaptation et Intégration Sociale, Université Laval, Quebec, Canada; 5 Department of Neuro Musculo Skeletal Lab, Institut de Recherche Expérimentale et Clinique, Secteur des Sciences de la Santé, UCLouvain, Brussels, Belgium

**Keywords:** patient outcome assessment, rehabilitation, physiotherapy modalities, evidence-based practice, Africa

## Abstract

**Background:**

The use of standardised assessment tools is a fundamental aspect of good clinical practice. However, to our knowledge, no study has documented the use of standardised assessment tools in physiotherapy in French-speaking sub-Saharan Africa.

**Objectives:**

Documenting the use of standardised outcome measures in physiotherapy in French-speaking sub-Saharan Africa.

**Method:**

Our cross-sectional survey used an online self-questionnaire on facilitators and barriers to the use of standardised outcome measures, distributed to physiotherapists in French-speaking sub-Saharan Africa.

**Results:**

A total of 241 physiotherapists working in French-speaking sub-Saharan Africa responded to the survey. The most represented countries were Benin (36.9%), Cameroon (14.1%), and Burkina Faso (10.8%). Although 99% of participants reported using standardised outcome measures, only 27% of the respondents used them systematically (all the time). The most reported facilitators included the recognition that standardised outcome measures help to determine whether treatment is effective, help to guide care, and improve communication with patients. The most significant barriers were the lack of time, unavailability of the standardised outcome measures, and non-sensitivity of measures to patients’ cultural and ethnic concerns. There was a higher proportion of use in the middle age group (30–40) (*p* = 0.02) and a lower proportion of use in physiotherapists simultaneously working in public and private sectors (*p* = 0.05).

**Conclusion:**

Standardised outcome measures are still not widely used by physiotherapists in French-speaking sub-Saharan Africa.

**Clinical implications:**

The perceived barriers and facilitators could help to develop strategies to improve the systematic use of outcome measures in French-speaking sub-Saharan Africa.

## Introduction

Standardised outcome measures enable *therapists* to quantify various aspects of a person’s functioning, such as impairments, activity limitations, participation, and quality of life (De Vet et al. 2011). These instruments can be used for diagnostic purposes, to support clinical decision making, to evaluate the effect of health care interventions, and to determine prognoses (Bausewein et al. 2018; Kostanjsek 2011).

As part of good clinical practice, physiotherapists worldwide have become more aware of the importance of adopting standardised outcome measures (Verheyden & Meyer 2016). Their use is an integral part of evidence-based practice, which is essential for optimal health care delivery (Copeland, Taylor & Dean 2008; Jette et al. 2003; Pattison et al. 2015; Potter et al. 2011). However, despite these recommendations, several studies which were, for the main part, conducted in high-income countries, have provided evidence that physiotherapists reported a limited use of standardised outcome measures. For instance, studies published decades ago reported proportions of outcome measures use ranging from 40% to 60% among physiotherapists of the Netherlands (Van Peppen et al. 2008), New Zealand (Copeland et al. 2008), United States (Jette et al. 2009), and Egypt (El-Sobkey & Helmy 2012). More recently, a Swedish study reported that all surveyed physiotherapists considered the use of outcome measure to be an important factor of good clinical practice and for all of them, these instrumental measures were available at their workplace (Käll, Larsson & Bernhardsson 2016). However, despite the existence of such knowledge and the availability of the materials, only 55% of the participants reported using outcome measures in the context of their practice (Käll et al. 2016). Another survey undertaken in Germany revealed that only 15% of the physiotherapists used outcome measures all the time, 31% frequently, 26% very rarely, and 14% did not use them at all (Braun et al. 2018).

Several barriers to the use of standardised outcomes measures have been reported. The most important barriers being the level of knowledge and skills of physiotherapists in using these instruments (Parry et al. 2015; Swinkels et al. 2011); structural limitations such as lack of time, unavailability of instruments, and lack of administrative support; and lack of consensus on which outcome measure to use for a given health condition and in a specific context (Demers et al. 2019; Duncan & Murray 2012; Gutiérrez Panchana & Hidalgo Cabalín 2018; Van Peppen et al. 2008).

In sub-Saharan Africa, very few studies have focused on the use of standardised outcome measures. The few studies that exist are mainly from English-speaking countries. In Nigeria, Akinpelu and Eluchie (2006) found a lower than 40% use of outcome measures by physiotherapists whatever the field of intervention (Akinpelu & Eluchie 2006). The authors reassessed 10 years later (in 2016) a representative sample of the participants to their first study, and noticed a slight improvement in the familiarity and use of the outcome measures but with a persisting higher percentage of non-use (over 60%) (Odole et al. 2018). However, later in 2019, Odole et al. (2019) reported contrasting results with a 68% use of knee osteoarthritis outcome measures among Nigerian physiotherapists (Odole et al. 2019). Agyenkwa et al. (2020) found that Ghanaian physiotherapists (47.6%) had low use of standardised outcome measures (Agyenkwa et al. 2020). One study from Nigeria dealt with harvesting perceived barriers and facilitators to the use of outcome measures (Obembe et al. 2019). The biggest perceived facilitators were familiarity with the tool (87.7%), positive attitude towards outcome measures (87.7%), and the objectivity of the measures (89.1%) (Obembe et al. 2019). The main perceived barriers were the need for additional accommodations to apply the outcome measures (63%) and the lack of time (44.2%) (Obembe et al. 2019).

To our knowledge, no studies have investigated the use of standardised outcome measures in French-speaking sub-Saharan Africa. Given the higher socio-economic levels with a more advanced level of physiotherapy development in English-speaking countries compared to French-speaking sub-Saharan Africa, the results of studies issued from English-speaking countries cannot be generalised to the whole of sub-Saharan Africa. Therefore, to meet *this need*, the aim of our study was to evaluate the use of standardised outcome measures in physiotherapy through the experiences, facilitators, and obstacles encountered by physiotherapists in French-speaking sub-Saharan Africa.

## Methods

Our cross-sectional online survey was addressed to physiotherapists practising in French-speaking sub-Saharan Africa. The survey consisted of a self-administered questionnaire developed on Google Forms. The homepage of the questionnaire provided a brief description of the aims of our study, the estimated time needed to complete all sections of the questionnaire, and some instructions on how to fill it in. The CROSS guidelines were followed for the reporting of the results (Sharma et al. 2021).

### Participants

We used a convenience sampling method to recruit physiotherapists from French-speaking sub-Saharan Africa (Benin Republic, Togo, Burkina Faso, Ivory Coast, Cameroon, Senegal, Mali, Niger, Democratic Republic of Congo, Burundi, the Central African Republic, Gabon, and Guinea). There were no exclusion criteria. According to data on the World Physiotherapy site, in 2022, there was an estimated 250 registered physiotherapists in Benin Republic, 400 in Togo, 190 in Ivory Coast, 250 in Cameroon, 66 in Niger, 1300 in the Democratic Republic of Congo, and 120 in Senegal (World Physiotherapy 2022). There were an estimated 50 physiotherapists in Burkina Faso, 25 in Burundi, and 10 in the Central African Republic, based on interviews with physiotherapists working in these countries. As this was an online survey, it was difficult to anticipate the rate of participation. However, we expected a participation rate of at least 10% of the targeted population.

### Development of the self-administered questionnaire

The questionnaire was designed based on previous studies aiming to assess facilitators and barriers to the use of standardised outcome measures (Al-Muqiren et al. 2017; Braun et al. 2018; Demers et al. 2019; Duncan & Murray 2012; Obembe et al. 2019; Odole et al. 2019). The questionnaire consisted of two sections. In the first section (12 items), physiotherapists were asked to provide personal and professional information, such as their country of residency, gender, age, areas of practice, number of years of professional experience, level of education, average number of patients treated per week, number of hours worked per week, average age of patients treated, number of physiotherapist colleagues practising in their centre, if applicable, and areas of intervention. The second section (11 items) concerned data related to the outcome measures. Participants were assessed on the frequency of patients’ assessment, the frequency of use of outcome measures according to a five-point Likert scale (all the time, most of the time, sometimes, rarely and never), facilitators and barriers to the use, and reasons guiding the choice of standardised assessment tools. Participants were also asked to name up to five of the most used outcome measures.

To ensure the comprehension and clarity of instructions and questions, the questionnaire was first pilot tested. This pre-test involved 10 physiotherapists (6 in Benin, 3 in Burkina Faso, and 1 in Ivory Coast). Based on the suggestions received, minor modifications to the phrasing of the questions were made to obtain a final version. The questionnaire took about 15–20 min to complete.

### Questionnaire diffusion

The Google form link of the questionnaire was sent to physiotherapists in sub-Saharan French-speaking Africa through social networks (*WhatsApp, Telegram, Messenger*) of their physiotherapy associations (*Association Béninoise des Kinésithérapeutes Réadaptateurs* [ABEKIR], *Association Sénégalaise des Kinésithérapeutes Rééducateurs* [ASKIR], *Rassemblement des Physiothérapeutes de l’Afrique Francophone* [RAPAF], *Société d’Afrique Francophone de Neurorehabilitation* [SAFNeR]), by e-mail invitations to the boards of physiotherapy associations (in the Democratic Republic of Congo, Ivory Coast, Togo, and Mali), to physiotherapy colleagues from countries that did not have associations at the time the study was conducted (Central Africa, Burkina-Faso, Burundi, and Chad). All colleagues and associations were requested to share the link of the questionnaire with their contacts in other French-speaking African countries. The Google form link was accessible with computer or smartphones. Reminder messages were sent every 2 weeks. Data collection began in April 2021 and ended in August 2021. A decision was made to end our study when no new responses were reported despite subsequent reminder messages.

### Statistical analysis

Data were collected in Microsoft Excel^®^ version 2016. Descriptive analyses were performed using the statistical software EPI INFO^®^ version 7.2.1.0. The chi-square (χ^2^) test was used to investigate associations between the frequency of use of standardised outcome measures and demographic factors. For that analysis, we grouped the response modalities ‘sometimes’ and ‘rarely’ into one ‘sometime or rarely’. The *p*-value was set at the significance level of α = 0.05. Analysis was based on available data for each variable of the questionnaire.

### Ethical considerations

All responding physiotherapists participated anonymously and voluntarily. All were informed that by completing the questionnaire they were giving their informed consent for the analysis and publication of the data.

## Results

A total of 241 physiotherapists from French-speaking sub-Saharan Africa participated in the study. The most represented countries were Benin (89 [36.9%]), Cameroon (34 [14.1%]), Burkina Faso (26 [10.8%]), and Togo (23 [9.5%]). The socio-demographic and professional characteristics are presented in Table 1. Men were the most represented (58%). Most respondents had a bachelor’s degree (76.3%), and more than half the participants (54.8%) had 5 years or less of professional experience.

**TABLE 1:** Socio-demographic and professional characteristics (*N* = 241).

Participant characteristics	Number	Percentage (%)
**Country**		
Benin	89	36.9
Cameroon	34	14.1
Burkina	26	10.8
Togo	23	09.6
Ivory Coast	16	06.6
Other	53	22.0
**Gender**		
Female	100	41.5
Male	141	58.5
**Age (years)**		
20–29	89	36.9
30–39	106	43.0
> 40	46	19.1
**Sector of activity**		
Public	81	33.6
Private	114	47.3
Public and private	46	19.1
**Years of professional experience**		
0–5	132	54.8
5–10	56	23.2
10–20	48	19.9
> 20	05	02.1
**Highest degree**		
Bachelor	184	76.3
Master	44	18.3
PhD	4	1.7
Other	9	3.7
**Number of patients treated per week**		
0–10	84	34.7
10–20	69	28.4
20–30	37	15.5
30–40	32	13.4
> 40	19	8.0
**Working hours per week**		
1–16	58	24.3
17–32	48	20.1
33–48	108	45.2
> 48	25	10.4
**Area of intervention**		
Neurology	56	23.2
Orthopaedics	41	17.4
Rheumatology or Traumatology	97	40.3
Paediatrics	11	4.6
Other	35	14.5

### Patient assessment and use of standardised outcome measures

Of the 241 physiotherapists surveyed, 52.3% reported that they assessed patients all the time but not necessarily using standardised outcome measures, 30.7% most of the time, 13.7% sometimes, 2.5% rarely, and 0.8% never.

As for the use of standardised outcome measures *during assessments*, of the 241 participating physiotherapists, 27% reported using standardised measures all the time, 35% used them most of the time, 25% used them occasionally, 12% used them rarely, and 1% never used them.

### Facilitators and barriers to the use of outcome measures

Reported facilitators and barriers to the use of standardised outcome measures are presented in Table 2 and Table 3, respectively. Among the provided response options, the most selected facilitators were: ‘the results or scores obtained with the outcome measures help to improve the effectiveness of treatment’ (65%), ‘the results or scores obtained with the outcome measures help to guide treatment’ (57%), and ‘I am convinced of the benefits of using the outcome measures’ (46%).

**TABLE 2:** Distribution of physical therapists according to the reasons facilitating the use of standardised outcome measures (*N* = 238).

Facilitators	*n*	Frequency (%)
Outcome measures are mandatory in my department	18	7.6
The results or scores obtained with the outcome measures help to improve the effectiveness of the treatment	155	65.1
I use outcome measures when there is administrative support	18	7.6
Outcome measures are easy to administer	25	10.5
The results or scores obtained with the outcome measures improve communication with patients	82	34.5
The results or scores from the outcome measures help to guide treatment	137	57.6
The results or scores obtained with the outcome measures help to motivate patients	71	29.8
There is no charge for the use of outcome measures; they are available and free of charge in my department	25	10.5
I am convinced of the benefits of using outcome measures	110	46.2
I receive financial compensation when I use the outcome measures	1	0.4
In my department, outcome measures are used on a daily basis	25	10.5
I use outcome measures when the patient cooperates well	34	14.3

**TABLE 3:** Distribution of physical therapists according to the reasons for not using standardised outcome measures (*N* = 171).

Barriers	*n*	Frequency (%)
The results or scores obtained with the outcome measures are difficult to interpret	12	7.1
Outcome measures are not freely available in my department	33	19.6
Outcome measures are not sensitive to patients’ cultural and ethnic concerns	38	22.6
Lack of administrative support	61	36.3
In my opinion, outcome measures are not useful	1	0.6
In my opinion, outcome measures do not help to guide the plan of care	6	3.6
Lack of time	82	48.8
Lack of financial compensation	11	6.5
Lack of knowledge	14	8.3
Outcome measures are not translated into the language spoken by the therapist and/or patient	34	20.2
I have difficulties in choosing the right outcome measure	21	12.5
The home care practice context does not favour the use of outcome measures	25	14.9
I do not use outcome measures because the patient does not cooperate	8	4.8

The main barriers to the routine use of outcome measures were the lack of time (48%), the lack of administrative support (36%), and the non-sensitivity of outcome measures to patients’ cultural and ethnic concerns (22%).

### Reasons for choosing standardised outcome measures

Validity (57%), reliability (73%), and the administration time (42%) were the reasons that most often guided therapists’ choice of using outcome measures (see Table 4).

**TABLE 4:** Distribution of physical therapists according to the reason for choosing standardised outcome measures (*N* = 236).

Reasons for choosing outcome measures	*n*	Frequency (%)
Validity of the outcome measure	136	57.1
Reliability of the outcome measure	174	73.1
Responsiveness to change of the outcome measure	80	33.6
The delay or time of administration of the outcome measure	102	42.9
The outcome measure is free of charge	57	23.9
Adaptability of the outcome measure to the (local) socio-cultural context	87	36.6
The availability of the outcome measure in the local language	37	15.5
Other	20	8.0

### Commonly used outcome measures

A total of 197 participants answered this question and 51 different outcome measures were named. The most frequent standardised outcome measures reported were Visual Analogue Scale (VAS, 60%), goniometer (39%), Functional Independence Measure (FIM, 31%), Lasegue test (29%), Functional Disability Scale for the Evaluation of Low Back Pain (FDSLP, 24%), 6-minute walk test (18%), manual muscle testing (18%), 10-m walk test (13%), and tape measure (11%). When the tools were grouped by category, the clinical tests (VAS, goniometer, Lasegue, Schoëber, walking tests, etc.) accounted for 72.72%, and the functional evaluation questionnaires (MIF, Dallas scale, Constant score) for 27.28%.

### Factors associated with the use of standardised outcome measures

Factors associated with the use of standardised outcome measures are summarised in Table 5. The variables country, gender, sector of activity, place of practice, number of years of experience, level of education, number of hours worked per week, age of patients, number of physiotherapist colleagues, and area of intervention were not statistically associated with the frequency of use of standardised outcome measures (*p* > 0.05). Only the age of the physiotherapists was significantly associated (*p* = 0.02) with a higher proportion of outcomes use in the middle age group (30 years – 39 years), and the area of activity was marginally significantly associated (*p* = 0.05) with less outcome use among physiotherapists who were simultaneously working in both public and private sectors.

**TABLE 5:** Participants’ characteristics associated with the use of outcome measures.

Participant characteristics	Frequency of use						Statistical analysis		
	All the time		Most of the time		Sometimes or rarely		χ^2^	*df*	*p*
	*n*	%	*n*	%	*n*	%			
**Country**	-	-	-	-	-	-	18.97	10	0.39
Benin	19	21.35	31	34.83	39	43.82	-	-	-
Burkina	7	26.92	7	26.92	12	46.16	-	-	-
Cameroon	11	32.35	14	41.18	9	26.47	-	-	-
Ivory Coast	5	31.25	4	25.00	7	43.75	-	-	-
Togo	10	43.48	9	39.13	4	17.39	-	-	-
Other	11	21.57	19	37.25	21	41.18	-	-	-
**Gender**	-	-	-	-	-	-	1.68	2	0.42
Male	40	26.37	52	36.88	49	34.75	-	-	-
Female	25	25.00	32	32.00	43	43.00	-	-	-
**Age (years)**	-	-	-	-	-	-	14.90	4	0.02
20–29	28	31.46	22	24.72	39	43.82	-	-	-
30–39	21	19.81	50	47.17	35	33.02	-	-	-
> 40	16	34.78	12	26.09	18	39.13	-	-	-
**Sector of activity**	-	-	-	-	-	-	9.34	4	0.05
Public	34	29.82	36	31.58	44	38.60	-	-	-
Private	26	32.10	25	30.86	30	37.04	-	-	-
Public and private	5	10.87	23	50.00	18	39.13	-	-	-
**Years of experience**	-	-	-	-	-	-	3.16	4	0.53
0–5	30	22.73	47	35.60	55	41.67	-	-	-
5–10	19	33.93	19	33.93	18	32.14	-	-	-
> 10	65	26.97	84	34.85	92	38.18	-	-	-
**Level of education**	-	-	-	-	-	-	7.43	4	0.11
Licence	42	22.83	67	36.41	75	40.76	-	-	-
Master	18	40.91	14	31.82	12	27.27	-	-	-
Other	5	38.46	3	23.08	5	38.46	-	-	-
**Working hours per week**	-	-	-	-	-	-	8.10	6	0.23
1–16	19	32.75	22	37.93	17	29.32	-	-	-
17–32	10	20.83	13	27.08	25	52.09	-	-	-
33–48	28	38.35	42	57.53	3	4.20	-	-	-
> 48	8	32.00	6	24.00	11	44.00	-	-	-
**Number of patients treated per week**	-	-	-	-	-	-	7.73	6	0.25
0–10	28	33.73	26	31.33	29	34.94	-	-	-
10–20	17	25.00	25	36.76	26	38.24	-	-	-
20–30	8	21.62	9	24.32	20	54.06	-	-	-
> 30	12	23.53	23	45.10	16	31.37	-	-	-
**Number of physical therapy colleagues**	-	-	-	-	-	-	7.81	8	0.45
0	9	21.43	12	28.57	21	50.00	-	-	-
1–2	15	30.00	14	28.00	21	42.00	-	-	-
3–5	19	27.14	30	42.86	21	30.00	-	-	-
6–10	11	30.55	10	27.78	15	41.67	-	-	-
> 10	11	26.83	17	41.46	13	31.71	-	-	-
**Area of intervention**	-	-	-	-	-	-	8.63	6	0.50
Neurology	17	30.36	17	30.36	22	39.28	-	-	-
Orthopaedics or Traumatology	11	26.19	16	38.09	15	35.72	-	-	-
Rheumatology	20	22.47	39	43.83	30	33.70	-	-	-
Paediatrics	4	36.36	3	27.28	4	36.36	-	-	-

## Discussion

Our study aimed to evaluate the systematic use of standardised outcome measures in French-speaking sub-Saharan Africa. A sample of 241 physiotherapists participated in our survey. Our results show that a large majority of physiotherapists used standardised outcome measures (99%) in their clinical practice, but few (only 27%) use them systematically (all the time). The main facilitators to the use of outcome measures were the potential to determine whether treatment is effective (65.1%), to guide treatment (57.6%), to improve communication with patients (34.5%), and being convinced of the benefits of using outcome measures (46.2%). The main barriers reported were the lack of time (48.8%) and administrative support (36.3%). The factors that contributed the most to the choice of an outcome measure over another were the knowledge of the psychometric qualities of the instrumental measures, including reliability (73.1%) and validity (57.1%), and the time required to administer the outcome measure (42.9%). Lastly, the age of physiotherapists and their sector of activity (public or private) were significantly associated with the frequency of use of assessment tools, with a higher proportion of use at middle age (30 years – 39 years) group, and low proportion of use among those simultaneously working in both public and private sectors.

The low systematic use of outcome measures among physiotherapists was also reported in a few studies conducted in sub-Saharan Africa. Indeed, in 2006, Akinpelu et al. reported only 40% use of outcome measures among Nigerian physiotherapists (Akinpelu & Eluchie 2006), which slightly improved 10 years later (in 2016) (Odole et al. 2018). Odole et al. also reported, about a dozen years later (in 2019), an increased proportion of use of outcome measures (67.5%) among physiotherapists working in the management of knee osteoarthritis (Odole et al. 2019). However, among these 67.5%, only 3.8% used outcome measures systematically (meaning all the time) and 26.9% often. The rest (36.8%) used them occasionally or rarely. In Ghana, Agyenkwa et al. (2020) also showed that 21% of physiotherapists working in stroke care used standardised outcome measures all the time (5/5 patients) and 21% often (3-4/5 patients) (Agyenkwa et al. 2020). Our results are similar to those of Demers et al. (2019) who compared the use of outcome measures between India and Canada. The authors reported a higher proportion of physiotherapists that frequently used (all the time and often) outcome measures in neurology (in both India and Canada), with 58% (about 18% all the time and 40% often) in India, and 56% (about 28% all the time and 28% often) in Canada. In a survey conducted in Saudi Arabia in 2017, it was also observed that most participants (62%) used standardised outcome measures in their practice (Al-Muqiren et al. 2017). Accounting for the results of these surveys, we acknowledge an evolution in the proportion of physiotherapists using outcome measures. This may be related to the increasing awareness of evidence-based practice in developing countries (Demers et al. 2019). However, although the use of outcome measures has globally increased over time, the proportion of physiotherapists that use them all the time remains low, despite current recommendations.

The main facilitators reported in our study were that the outcome measure could help with monitoring treatment effectiveness, could help to guide treatment, could improve communication with patients, and being convinced of the benefits of using outcome measures. These findings are similar to findings from Nigerian studies (Obembe et al. 2019; Odole et al. 2019). Other facilitators reported in those studies not present in our study were the familiarity with the outcome measures, and a positive attitude towards these (Obembe et al. 2019; Odole et al. 2019). A Ghanaian study also reported that the availability of outcome measures and the recommendation for their use in the department were key facilitators (Agyenkwa et al. 2020). In the study by Demers et al. (2019), the main facilitators were the validity and reliability of the instrumental measure, also recognised in our study as a criterion for tool choice; the recommendation of the measure in current guidelines; learning to use the outcome measure during training, which is similar to the familiarity with the tool in the Nigerian studies; and the ease of administration and availability of the measure (Demers et al. 2019). In an American survey, about 90% of participants reported that using outcome measures improved communication between patients and therapists and also helped determine a plan of care (Jette et al. 2009), which is consistent with the observations in our study.

The main barriers we reported were the lack of time and administrative support. Similarly, all studies previously published reported that the lack of time was the main barrier to using outcome measures (Demers et al. 2019; Jette et al. 2009; Odole et al. 2019; Östhols et al. 2019; Renteria & Berg 2019). This justifies the fact that the time of administration was one of the main reasons that motivated the choice of outcome measures, as we observed. Another barrier reported was the non-adaptation of outcome measures to the socio-cultural context, which is mostly the case for measures involving latent, behavioural variables. This underlines the need for contextualisation studies regarding the use of outcome measures in Africa (Kossi et al. 2020; Sogbossi et al. 2014, 2022). However, while some standardised outcome measures have been adapted to the socio-cultural realities or translated and validated into local languages in both French- and English-speaking sub Saharan Africa countries (Sogbossi et al. 2014, 2022; Van Zyl et al. 2023), studies on how this improves their use in clinical practice are lacking. One could hypothesise that these outcome measures, although validated, have some cost implications related, for instance, to the availability of the materials or the training of professionals, resulting in no improvement in their use. Other barriers reported in Nigeria were the lack of motivation, the non-availability of outcome measures at the workplace, and the lack of financial support (Obembe et al. 2019; Odole et al. 2019). In addition to the lack of time, Demers et al. (2019), also reported the following barriers: the cost of the measures (more reported in India), the non-availability of the instruments (more reported in Canada), and a general lack of knowledge (more reported in India) (Demers et al. 2019). These results highlight some differences between countries in the use of outcome measures, which will need to be taken into consideration for the development of strategies aiming to improve their use.

These reported barriers and facilitators could partly contribute to the fact that most outcomes measures mentioned were clinical tests generally learnt during training (VAS, goniometer, tape measure, Lasegue, Neer diagnostic tests) which take less time, do not require materials, and are more familiar to physiotherapists. Akinpelu et al (2006) also observed that VAS was the most familiar outcome measure, as reported by Nigerian physiotherapists (Akinpelu & Eluchie 2006). However, these results could be influenced by the fact that more than half of our sample were working in the rheumatology and/or traumatology fields where pain measures and diagnostic tests are more likely to be used. Contrastingly, in the field of neurology, the Stroke Impact Scale was the most commonly used among adults with stroke in Ghana (Agyenkwa et al. 2020), and the Gross Motor Function Measure the most commonly used among children with cerebral palsy in Nigeria (Obembe et al. 2019).

Our results showed that there was a statistically significant relationship between the age of physiotherapists and the frequency of use of outcome measures. We reported a low rate of use among physiotherapists in the age categories of 20–29 as well as 40 and more years old. Regarding the 40 and older group, results could be explained by the fact that older physiotherapists are more likely to work on the basis of their experience; therefore, reducing their use of standardised outcome measures. It could also be explained by the fact that at the time of their training there was less awareness and knowledge about the use of standardised outcome measures. These results are similar to those of Agyenkwa et al. (2020) in Ghana who reported a significant higher use of outcome measures in the age group less than 40 compared to the group 40 and above (Agyenkwa et al. 2020). As for younger physiotherapists (20–29 years old), the low use of standardised outcome measures may be explained by the lack of clinical experience, while physiotherapists aged 30–39 years old, that is those in the middle of their careers, have had time to realise the added value of using standardised assessments to facilitate patient follow-up and improve clinical decision making.

Our results also showed a borderline statistically significant relationship between the sector of activity and the frequency of use of standardised outcome measures. Indeed, physiotherapists who work in both the public and private sectors tend to have a lower rate of use of outcome measures. One hypothesis from the literature to explain these results is that people working in both the public and private sectors may have less time to consult the guidelines regarding the importance of using standardised outcomes measure in clinical practice (Agyenkwa et al. 2020; Duncan & Murray 2012).

We expect that the results discussed so far will help in finding strategies to enhance the use of standardised outcome measures by physiotherapists and every rehabilitation professional in general, in French-speaking sub-Saharan Africa and beyond. In that perspective, Antunes et al. (2014) have proposed a five-step intervention strategy in a palliative care unit, that could be adapted: (1) selection of outcomes of interest, (2) selection of outcome measure(s), (3) educational component about measure and how to use results, (4) selection of one coordinator or facilitator, and (5) defining who applies the measure and its periodicity (Antunes et al. 2014). A recent systematic review on interventions to increase the use of standardised outcome measures by rehabilitation professionals, has also underlined the probable positive effect of educational training or workshops on selected outcome measures (Colquhoun et al. 2017). Moreover, the intervention strategies should address the specific barriers and facilitators of each clinical setting (Eilayyan et al. 2020).

### Limitations of our study

This is the first study to examine the use of standardised outcome measures among physiotherapists in French-speaking sub-Saharan Africa. However, some limitations must be acknowledged. Firstly, the survey was available only online. This could have increased the participation rate of physiotherapists familiar with digital media and online content. This may also have led to the omission of potential respondents working in areas without internet access and discouraged therapists who may be less comfortable with online content. Secondly, physiotherapists associations were the main channels of participants’ recruitment. As such, physiotherapists who were non-members of the associations might have missed the opportunity to participate in our study. Thirdly, although rigorous pilot testing was conducted for our questionnaire, formal reliability and validity assessments could have been undertaken to increase and ensure the overall quality of our work. Moreover, studies from Ghana (Amuasi et al. 2022) and Nigeria (Onyeso et al. 2022) had reported 16% and 19% rate of online survey participation from health-workers and physiotherapists, respectively, while in our study we reached nearly 10% of the targeted population. Because of the limited sample of respondents in some countries, our results could not be fully generalised to all French-speaking countries of sub-Saharan Africa. Further studies with a more representative sample of these less represented countries are needed to confirm the results of our survey.

## Conclusion

The use of standardised outcome measures in the evaluation and follow-up of patients is important to deliver quality interventions. While most physiotherapists of French-speaking sub-Saharan Africa use standardised outcome measures in clinical practice, very few (around a quarter) use them on a regular basis. The identified barriers and facilitators could help to develop strategies to improve the routine use of standardised outcome measures.
